# Immunophenotypic Implications of Reverse-Circadian Glucocorticoid Treatment in Congenital Adrenal Hyperplasia

**DOI:** 10.3390/ijms26041479

**Published:** 2025-02-10

**Authors:** Hanna F. Nowotny, Hannah Choi, Selina Ziegler, Natalie Doll, Ariane Bäuerle, Ann-Christin Welp, Ilja Dubinski, Katharina Schiergens, Uta Neumann, Lea Tschaidse, Matthias K. Auer, Simon Rothenfusser, Heinrich Schmidt, Nicole Reisch

**Affiliations:** 1 Department of Medicine IV, LMU University Hospital, LMU Munich, 80336 Munich, Germany; 2 Department of Pediatrics, Dr. von Hauner Children’s Hospital, LMU University Hospital, LMU Munich, 80336 Munich, Germany; 3 Klinik für Pädiatrische Endokrinologie und Diabetologie, Charité Universitätsmedizin Berlin, 13353 Berlin, Germany; 4 Division of Clinical Pharmacology, LMU University Hospital, LMU Munich, 80337 Munich, Germany; 5 Einheit für Klinische Pharmakologie (EKLiP), Helmholtz Zentrum München-German Research Center for Environmental Health Neuherberg, 85764 Oberschleißheim, Germany

**Keywords:** circadian, glucocorticoids, immunophenotype, congenital adrenal hyperplasia

## Abstract

Classic congenital adrenal hyperplasia due to 21-hydroxylase deficiency (CAH) requires lifelong glucocorticoid replacement to manage cortisol deficiency and excessive androgen production. Conventional circadian treatment (CT) tries to mimic natural cortisol rhythms, whereas reverse-circadian treatment (RC) prioritizes the suppression of adrenal androgen excess overnight through evening dosing. Limited data exist on the immunological impact of these regimens. A bi-centric study was conducted, including 41 pediatric and adolescent CAH patients. Peripheral blood samples were collected from patients on conventional treatment (*n* = 38) or RC (*n* = 16), with 11 RC patients switching to conventional treatment. Immune cell phenotypes, cytokine profiles, and natural killer (NK) cell cytotoxicity were assessed. Patients receiving RC showed lower percentages of CD4+CD25+ T cells (*p* = 0.0139). After the switch, patients with RC presented with a higher percentage of non-classical monocytes (*p* = 0.0255) and a lower percentage of Th17 cells (*p* = 0.0195). A lower expression of CD107 was observed with RC (*p* < 0.0001), as well as a higher percentage of NKp30 (*p* = 0.0189). Comparing patients after the switch from RC to HC, patients with RC presented with a lower NKG2D expression (*p* = 0.0420). Both conventional treatment and RC exhibited distinct immunological impacts, with CT showing modest advantages in normalizing immune phenotypes. These findings suggest that CT may offer immunological benefits for managing young patients with congenital adrenal hyperplasia.

## 1. Introduction

In healthy individuals, cortisol levels follow a distinct circadian rhythm, with the lowest concentrations at night, a consistent rise in the early morning, a peak on waking, and then declining concentrations during the course of the day [1]. Congenital adrenal hyperplasia (CAH) encompasses a group of inherited disorders characterized by enzymatic defects in adrenal steroidogenesis, leading to cortisol deficiency [2]. In the most common form, CAH due to 21-hydroxylase deficiency, diminished cortisol production leads to increased production of adrenocorticotropic hormone (ACTH) by the adrenal gland, thereby resulting in the overproduction of adrenal precursor steroids and adrenal androgens [3]. Management of the classic disease relies on lifelong glucocorticoid (GC) and mineralocorticoid replacement to prevent adrenal crises and mitigate excessive androgen production [2]. Standard regimens typically involve hydrocortisone (HC) administration in three daily doses, with the highest dose in the morning to align with the physiological diurnal cortisol rhythm. In this study, we will refer to the approach with circadian treatment (CT). This approach tries to mirror the natural peak of hypothalamic-pituitary-adrenal (HPA) axis activity in the morning, optimizing disease control and minimizing adverse effects [4]. However, alternative dosing strategies, such as reverse-circadian treatment (RC), which emphasizes higher evening doses to suppress the early morning HPA surge, have been explored and are preferred by some centers [5,6,7,8].

Evidence supporting RC remains mixed. A retrospective analysis of salivary 17-hydroxyprogesterone profiles of patients receiving either circadian or RC treatment showed no differences in the 17-OHP profiles of the two subgroups. They also raise concerns about potential long-term risks associated with RC, including overweight status, and suggest conventional circadian treatment, as RC lacks significant advantages in biochemical control [9]. Randomized trials comparing high-morning versus high-evening HC dosing in children with classical CAH have shown similar levels of morning androgen markers, sleep quality, and daytime activity between regimens, favoring the traditional high-morning dose approach [10,11]. Studies on the effect of GC treatment on sleep patterns [12,13,14] as well as nocturnal blood pressure [15] further suggest superiority of standard circadian treatment. However, no long-term outcome data for either regimen are available. Individual variability in treatment responses underscores the need for personalized dose distribution and frequent time and dose-dependent monitoring to achieve optimal outcomes.

Despite the focus on biochemical control, the broader physiological implications of GC replacement regimens remain underexplored, particularly their impact on immune function. GCs are key regulators of immune activity, and disruptions in their circadian dynamics—whether endogenous or treatment-induced—can profoundly influence immune phenotypes. For instance, adrenal insufficiency (AI) patients often exhibit increased susceptibility to infections and immune cell dysregulation [16,17]. Specific immune cell alterations, such as reduced natural killer (NK) cell cytotoxicity and T-cell subset imbalances, highlight the effects of GC treatment as well as further disease-specific effects in patients with PAI on immune function [18].

The relevance of understanding these immune alterations is underscored by the increased risk of adrenal crises in CAH patients [19]. This increased risk of adrenal crises correlates with a higher mortality rate compared to healthy controls, with increased hazard ratios of 2–3 [20]. These data suggest that, beyond immediate biochemical control, refining GC regimens to optimize immune function could have significant implications for long-term patient health and outcomes.

Despite these findings, significant knowledge gaps remain regarding how different GC regimens modulate immune responses over time and how they impact clinical outcomes such as infection rates or autoimmune predisposition. While studies have demonstrated immune dysregulation in AI patients, the specific immune consequences of RC versus circadian GC treatment in CAH remain poorly defined.

Modified-release hydrocortisone (MR-HC) offers a promising alternative to traditional regimens, aiming to restore physiological cortisol rhythms [21]. Clinical studies suggest that MR-HC normalizes immune cell profiles, reduces recurrent infections, and mitigates metabolic disturbances, such as glucose intolerance linked to evening cortisol elevations [22,23]. Furthermore, modified-release treatments may even better preserve clock gene expression and align immune phenotypes closer to those of healthy controls compared to conventional circadian treatment [24]. By reducing disruptions to immune regulation, these approaches might offer a pathway to improved health outcomes in CAH and other forms of AI.

In this study, we focus on the impact of circadian treatment (both conventional GC treatment and MR-HC treatment) vs. RC in a cohort of pediatric and adolescent patients with CAH on immunophenotype and function.

## 2. Results

### 2.1. Patient Characteristics

In the initial phase of the study, two cohorts of pediatric and adolescent patients with CAH receiving either circadian treatment or RC were analyzed for differences in their immunophenotypic profiles. The RC cohort included 16 patients (25% female, 75% male), while the circadian treatment cohort (*n* = 38) had a gender distribution of 45% females and 55% males. The median age was comparable between the cohorts (RC: 15 years; circadian treatment: 18 years; *p* = 0.0779). Similarly, there was no significant difference in BMI between the groups (RC: 24.07 kg/m^2^; circadian treatment: 23.68 kg/m^2^; *p* = 0.6136). Although the hydrocortisone dose equivalent (HDE) did not significantly differ between the two groups, HDE normalized to body surface area (HDE/BSA) was slightly lower in the circadian treatment cohort compared to the RC cohort (RC: 17.36 mg/day/m^2^; circadian treatment: 14.46 mg/day/m^2^; *p* = 0.0376). The demographic and clinical data are summarized in Table 1.

In the second part of the study, a subgroup of patients receiving RC was transitioned to circadian treatment using dose-equivalent adjustments. This subgroup included 8 male and 3 female patients, with ages ranging from 10 to 20 years. Dose-equivalent switching was generally well-tolerated, with up- or down-titration of ±2.5 mg/day required in only 2 of the 11 cases. Refer to Table 2 for more details regarding treatment doses.

### 2.2. Lymphocyte Populations in Pediatric and Adolescent Patients with CAH on Either Reverse-Circadian or Circadian Glucocorticoid Replacement Regimens

The flow cytometric analysis of B, T, and NK cell subsets, as well as monocytes, revealed no significant differences in the percentage of B-cells between the RC and circadian treatment groups (*p* = 0.2010). Similarly, the proportions of all three monocyte subsets—classical (*p* = 0.5730), intermediate (*p* = 0.3409), and non-classical (*p* = 0.1117)—were comparable between the treatment groups. No significant differences were observed in the total populations of CD3^+^ T cells, CD4^+^ helper T cells (Th1 [IFN-γ^+^], Th2 [IL-4^+^], Th9 [IL-9^+^], Th17 [IL-17^+^], and Th22 [IL-22^+^]), or CD8^+^ cytotoxic T cells (Tc1, Tc2, Tc9, Tc17, and Tc22).

Interestingly, the percentage of CD4^+^CD25^+^ T cells was significantly lower in the RC cohort compared to the circadian treatment cohort (RC: 4.990%; circadian treatment: 6.780%; *p* = 0.0138). However, Foxp3 expression within the CD4^+^CD25^+^ T-cell subset did not differ significantly between the two groups (*p* = 0.8470). The data are summarized in Table 3.

### 2.3. Lymphocyte Populations in Pediatric and Adolescent Patients with CAH Switched from Reverse-Circadian to Circadian Glucocorticoid Replacement Treatment

The flow cytometric analysis of B, T, and NK cell subsets, as well as monocytes, showed no significant differences in the percentage of B-cells before and six weeks to three months after the switch from RC to circadian treatment (*p* = 0.1230). Similarly, the proportions classical (*p* = 0.8467) and intermediate (*p* = 0.9102) monocytes remained comparable between patients receiving RC and after the switch to circadian treatment, while the percentage expression of non-classical monocytes appeared significantly higher in the reverse-circadian subgroup (RC: 26.00%; circadian treatment: 18.70%; *p* = 0.0255). No significant changes were observed in the total populations of CD3^+^ T cells, CD4^+^ helper T cells (Th1 [IFN-γ^+^], Th2 [IL-4^+^], Th9 [IL-9^+^], and Th22 [IL-22^+^]), or CD8^+^ cytotoxic T cells (Tc1, Tc2, Tc9, Tc17, and Tc22).

However, the percentage of Th17 cells significantly increased following the switch to circadian treatment (RC: 0.45%; circadian treatment: 0.46%; *p* = 0.0195). The previously observed difference in CD4^+^CD25^+^ T cells in the larger cohort was not evident in this subgroup analysis of patients directly switched from RC to circadian treatment. Foxp3 expression also remained unchanged before and after the medication regimen switch. Please refer to Table 4 for further information.

### 2.4. NK Cell Surface Phenotype and Function of Pediatric and Adolescent Patients with CAH Under Reverse-Circadian vs. Circadian Glucocorticoid Replacement Treatment

The total percentage of NK cells, including the subsets CD56brightCD16dim and CD56dimCD16bright, showed no significant differences in either the initial group comparison or in patients directly switched from RC to circadian treatment (Table 3 and Table 4). The analysis of the NK cell surface phenotypes revealed a lower expression of the degranulation marker CD107a in patients receiving RC compared to those receiving circadian treatment (RC: 45.05%; circadian treatment: 58.70%; *p* < 0.0001). Conversely, NKp30 expression was higher in the RC group than in the circadian treatment group (RC: 46.45%; circadian treatment: 33.10%; *p* = 0.0189; Table 3).

In the subgroup of patients directly switched from RC to circadian treatment, only NKG2D expression showed a significant difference (RC: 16.60%; circadian treatment: 23.40%; *p* = 0.0420; Table 4).

**Figure 1:**
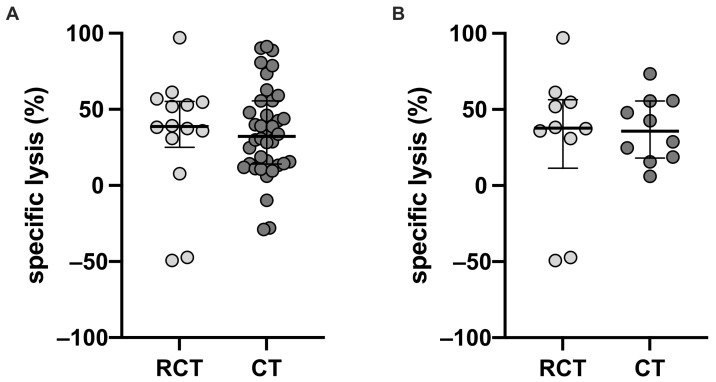
NK cell cytotoxicity of (**A**) pediatric and adolescent patients with CAH on either reverse-circadian (*n* = 14) or circadian (*n* = 38) glucocorticoid replacement regimens and (**B**) pediatric and adolescent patients with CAH on reverse-circadian treatment (*n* = 10) as well as after the switch to circadian (*n* = 10) GC replacement. Mann–Whitney test was used for the unpaired comparison, and the Wilcoxon test for the paired analysis, with *p* < 0.05 as statistically significant.

Despite differences in the NK cell surface marker expression, NK cell functionality remained unaffected. NK cell cytotoxicity (NKCC), measured as the specific lysis of the tumor cell line K562, was comparable between the two groups (Figure 1). At an effector-to-target cell ratio of 50:1, specific lysis was 38.75% (IQR: 25.14–55.27) in patients receiving RC and 32.30% (IQR: 14.03–55.57) in patients receiving circadian treatment (*p* = 0.6171; Figure 1A). Similarly, in patients switched from RC to circadian treatment, specific lysis remained comparable (RC: 37.79% [IQR: 11.38–56.36]; circadian treatment: 35.69% [IQR: 18.01–55.57]; *p* = 0.8457; Figure 1B).

### 2.5. Cytokine Profiles of Pediatric and Adolescent Patients with CAH Under Reverse-Circadian vs. Circadian Glucocorticoid Replacement Treatment

**Figure 2:**
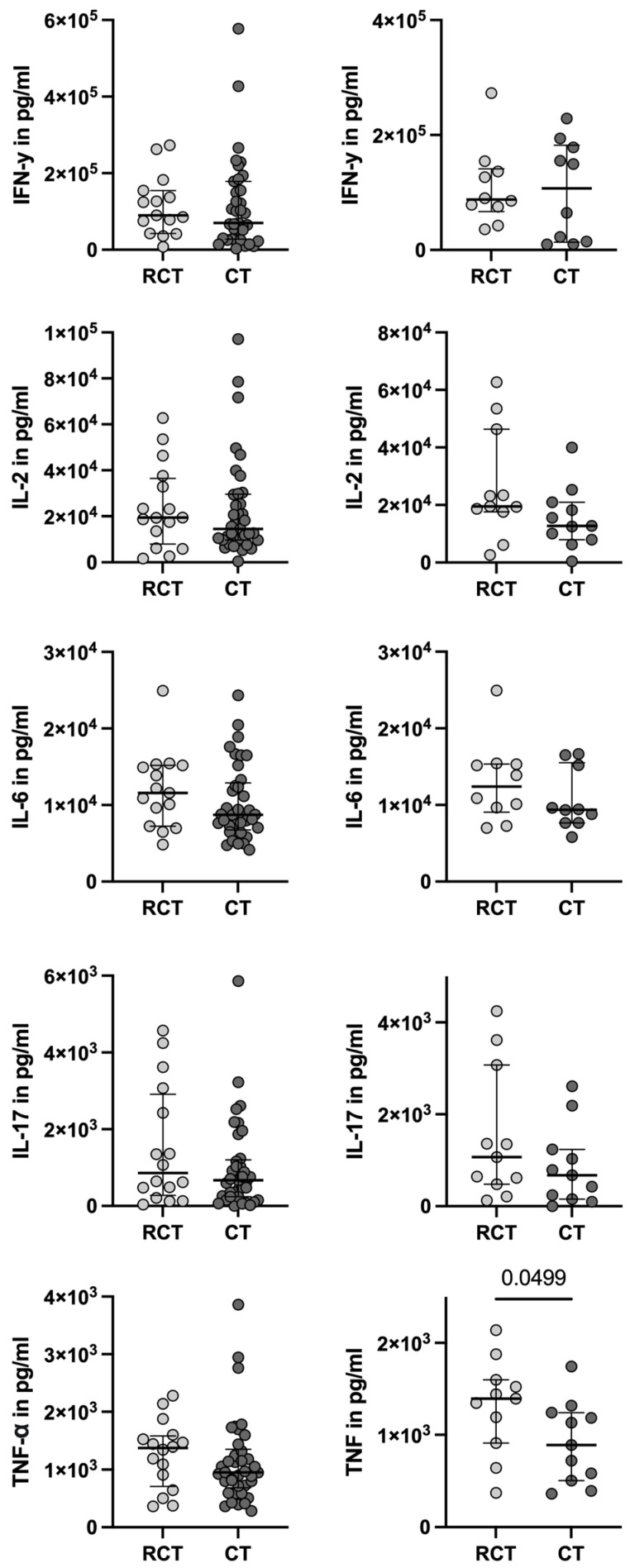
Measurements of cytokines IFN-γ, IL-2, IL-6, IL-17 and TNF-α after 24 h of stimulation of whole blood with LPS or PMA/Ionomycin. Depicted is the median concentration in pg/mL and IQR. Mann–Whitney test was used for the unpaired comparison, and the Wilcoxon test for the paired analysis, with *p* < 0.05 as statistically significant.

Cytokine expression following stimulation with either lipopolysaccharide (LPS) or phorbol myristate acetate (PMA)/ionomycin showed comparable responses for Interferon-γ (IFNγ), Interleukin-2 (IL-2), IL-6, and IL-17. However, Tumor Necrosis Factor-α (TNF-α) release was significantly higher in patients receiving RC compared to after the switch to circadian treatment (RC: 1395 pg/mL [IQR: 910–1599]; circadian treatment: 888.8 pg/mL [IQR: 506–1241]; *p* = 0.0499; Figure 2).

## 3. Discussion

In this bi-centric study, we analyzed immunophenotypic changes associated with circadian vs. reverse-circadian GC replacement regimens in pediatric and adolescent patients with congenital adrenal hyperplasia (CAH). While RC was associated with a slightly lower percentage of regulatory CD4+CD25+ T cells and higher TNF-α levels compared to circadian treatment, many other immune parameters, including NK cell functionality and most lymphocyte subsets, showed no significant differences. The switch from RC to circadian treatment resulted in minor changes, such as a modest increase in Th17 cells, but did not reveal profound shifts in immune profiles. These findings suggest that, in this small cohort, the timing of GC administration may have nuanced effects on immune modulation, but its clinical relevance requires further investigation to determine whether these subtle differences translate into meaningful outcomes.

To date, only a few studies have examined Treg expression and function in patients with primary adrenal insufficiency, reporting either a numerical downregulation of Foxp3^+^ Tregs or no significant changes in Treg expression [18,25]. CD4+CD25+ T cells, which include Tregs, play a crucial role in maintaining immune homeostasis and suppressing excessive inflammatory responses. The significantly lower percentage of CD4+CD25+ T cells in the RC cohort suggests that this regimen may impair immune regulation to some extent, potentially increasing susceptibility to inflammatory or autoimmune processes [26]. However, the lack of significant differences in Foxp3 expression within this subset suggests that the functional properties of Tregs may remain intact, indicating that this finding is unlikely to have substantial clinical relevance [27].

Similarly, Th17 cells are known to promote inflammatory responses and support mucosal immunity, yet their role in adrenal insufficiency remains underexplored. In this study, the modest increase in Th17 cells observed after switching from RC to circadian treatment likely represents a rebalancing of immune dynamics rather than a clinically significant change. Notably, previous studies have reported no differences in Th17 expression between patients with CAH and healthy controls [18], potentially due to the well-documented resistance of Th17 cells to the inhibitory effects of glucocorticoids [28]. Given the small magnitude of the observed increase following the switch and the lack of associated shifts in inflammatory markers or clinical outcomes, this finding is unlikely to have substantial clinical relevance.

The observed differences in NK cell surface marker expression, such as reduced CD107a and increased NKp30 in the RC cohort, alongside increased NKG2D following the switch from RC to circadian treatment, highlight potential alterations in NK cell activation states influenced by GC timing. CD107a is a marker of degranulation and cytotoxic activity, suggesting that its lower expression in the RC group may indicate a subdued activation state of NK cells [29]. Conversely, higher NKp30 expression in the RC cohort might reflect compensatory upregulation of activation receptors [30]. The increase in NKG2D following the transition to circadian treatment aligns with enhanced recognition of stress-induced ligands, potentially improving NK cell-mediated immune surveillance [31]. Despite these shifts in surface marker expression, NK cell cytotoxicity, as measured by specific lysis of target cells, remained preserved across treatment regimens. These results suggest that while GC timing may influence certain phenotypic characteristics of NK cells, the core functionality of these immune cells remains robust, irrespective of the changes in surface activation markers, minimizing the clinical impact of observed marker variations.

The elevated TNF-α levels observed in patients on RC and the reduction seen after switching to circadian treatment suggest that RC may induce a more pro-inflammatory state. TNF-α is a key cytokine involved in inflammation, and its elevated levels could disrupt immune regulation in CAH patients [32]. The decrease in TNF-α following the switch to circadian treatment indicates that aligning GC dosing with the natural circadian rhythm may help reduce inflammation and support immune balance. These findings underscore the potential benefits of circadian treatment in minimizing chronic inflammation and optimizing immune function in CAH patients.

In evaluating the relative merits of circadian treatment and RC regarding immune phenotype in patients with CAH, our study observed some minor immune changes, but no substantial improvements in immune function with RC. Due to the small sample size and limited observation period, we are unable to assess clinical outcomes such as infection rates in these cohorts. However, the minor changes in immune cell subsets observed with circadian treatment support its potential benefits in immune regulation, as it more closely resembles healthy immune phenotypes. This alignment may help reduce the risk of infections and immune dysregulation, suggesting that circadian treatment could be a more effective approach for maintaining long-term health in CAH patients by lowering the increased mortality rate associated with infection-related adrenal crises [20]. Preliminary data also suggest additional benefits of MR-HC treatment, which more accurately mimics the physiological circadian release of cortisol, resulting in fewer alterations to the immune cell profile compared to conventional circadian treatment [33].

While the dose differences between morning and evening administrations were relatively small—sometimes as little as 2.5 mg—observable trends were still noted. Reversing the dosing ratio to 2/3 in the morning and 1/3 in the evening might produce more pronounced effects. It is also important to emphasize that this is a real-life study and that other centers also administer long-acting formulations and higher doses.

This study has several limitations that should be considered when interpreting the results. Firstly, the sample size, particularly in the subgroup analyses, was relatively small, which may limit the generalizability of our findings and the statistical power to detect significant differences. Additionally, the short-term follow-up for patients who switched from RC to circadian treatment may not fully capture the long-term effects of these regimens on immune function and overall health. Another key limitation is the lack of comprehensive data on clinical outcomes, such as infection rates and quality of life, which are crucial for understanding the real-world impact of different glucocorticoid regimens in CAH patients. Furthermore, patient assignment to the RC or CT groups was based on clinician preference and subjective patient preference rather than a standardized protocol, which may introduce selection bias. Similarly, the reasons for switching from RC to CT were determined by individual clinical considerations, potentially influencing observed outcomes. These factors should be taken into account when interpreting the findings of this study.

Our data build on previously published evidence, demonstrating that neither treatment shows superiority in patients with CAH regarding biochemical control or immunological phenotype and function in the short term. Beyond the lack of significant biochemical advantages over circadian treatment, RC has been associated with increased metabolic risks, worsening nocturnal blood pressure, and disrupted sleep patterns [9,10,11,12,13,14,15]. Our data now suggest that circadian treatment may offer benefits in preserving immune function and reducing inflammatory markers in children and adolescents with CAH even at low replacement doses and as short-term effects, although the changes observed after directly switching from RC to circadian treatment were modest. Approaches like MR-HC treatment may offer potential for further refinement of treatment outcomes, also regarding immune status.

## 4. Materials and Methods

### 4.1. Subjects

In this bi-centric study we included 41 children and adolescents with classic CAH due to 21-hydroxylase deficiency. All the patients were recruited from the Endocrine Outpatient Clinic of the University Hospital Munich, from the endocrine outpatient clinic of the Dr. von Haunersche Children’s Hospital of the University Hospital Munich, as well as from the Department of Pediatric Endocrinology and Diabetology of the Charité University Berlin. Of these 41 patients, 38 samples were collected of patients receiving a circadian replacement regimen with either conventional TD hydrocortisone substitution (HC, *n* = 18) or modified-release hydrocortisone (MRHC, *n* = 20). Additionally, 16 patients with a reverse-circadian hydrocortisone regimen (RC) were included in the study. Of the 16 patients on RC, 11 patients were dose-equivalently (±2.5 mg hydrocortisone dose equivalent (HDE)) switched to a circadian regimen for clinical reasons. Blood samples were collected from all patients at baseline and, for those applicable, six weeks to three months after the switch of medication. The inclusion criteria comprised a genetically confirmed diagnosis of classic congenital adrenal hyperplasia (CAH) due to 21-hydroxylase deficiency, an age up to 22 years, and stable glucocorticoid (GC) therapy (type and dose) for a minimum of three months. Patients with acute infections, malignancies, shift-work schedules, or pregnancy were excluded from the analysis. All patients gave written informed consent to participate in our registry and biobank for adrenal insufficiency and differences in sex development (Bio AI/DSD, ethical approval no. 19–558). Patient samples were collected during routine follow-up visits after intake of their morning medication.

### 4.2. Isolation of Immune Cells

Blood samples were collected across all four seasons from each of the analyzed patient subgroups. Heparinized blood was processed within 12 h of collection to isolate peripheral blood mononuclear cells (PBMCs) and minimize apoptotic effects. For each patient, three 7.5 mL tubes of heparinized blood were collected, and PBMCs were isolated using a standardized density centrifugation protocol. The isolated cells were cryopreserved in 10% dimethyl sulfoxide (DMSO, Sigma-Aldrich^®^, Burlington, MA, USA) and fetal calf serum (FCS, Thermo Fisher Scientific^®^, Waltham, MA, USA). Cryopreservation was performed at −80 °C using an isopropanol freezing chamber (Mr. Frosty^®^, Sigma-Aldrich^®^) before subsequent analysis by multicolor flow cytometry. Additionally, 400 µL of heparinized blood was diluted 1:4 in RPMI medium supplemented with 2 mM L-glutamine, 100 U/mL penicillin, and 100 µg/mL streptomycin. Triplicates were either left unstimulated or stimulated for 24 h with LPS (eBioscience™ LPS solution (500X), diluted to a final concentration of 100 ng/mL, Invitrogen™, Carlsbad, CA, USA) or PMA/ionomycin (eBioscience™ Cell Stimulation Cocktail (500X), Invitrogen™, diluted 1/500 to a final concentration of 2 µL/mL). After incubation, supernatants were collected, pooled in triplicates, and stored at −80 °C for subsequent cytokine profiling via sandwich ELISA.

### 4.3. Thawing and Staining of PBMCs

Cryopreserved PBMCs were thawed in a 37 °C water bath and washed in complete medium (450× *g*, room temperature, 10 min). The cells were then incubated overnight at 37 °C with 5% CO_2_ to allow recovery. Prior to staining, the samples were stimulated for four hours using phorbol myristate acetate (PMA)/ionomycin (eBioscience™ Cell Stimulation Cocktail, diluted 1:500 to a final concentration of 2 µL/mL, Invitrogen™). To block nonspecific binding sites, Human TruStain FcX^®^ (BioLegend^®^, San Diego, CA, USA) was applied. Subsequently, the cells were stained with anti-human antibodies or their respective isotype controls (detailed panels below) for 30 min at 4 °C, using a concentration of 0.7 µL per 100 µL. For intracellular staining specific to the T-cell panel, the eBioscience™ Foxp3/Transcription Factor Staining Buffer Set (Invitrogen^®^) was utilized according to the manufacturer’s instructions. Stimulation was again performed with PMA/ionomycin, and Brefeldin A (Invitrogen^®^) was added as a protein transport inhibitor. Cells were fixed with 4% paraformaldehyde (PFA) and subsequently transferred to Dulbecco’s Balanced Salt Solution (DPBS) for multicolor flow cytometry analysis.

### 4.4. Assessment of Leucocyte Subsets and Surface Phenotypes

We performed an analysis of immune cell subsets via multicolor flow cytometry. The first antibody panel included CD45-BV605 (Clone HI30), CD3-PECy5 (Clone HIT3), CD8-PECy7 (Clone RPA-T8), CD4-PerCPCy5.5 (Clone OKT4), CD19-BV421 (Clone HIB19), CD56-FITC (Clone HCD56), CD14-BV510 (Clone M5E2), CD16-AF647 (Clone 3G8), Granzyme B-AF647 (Clone GB11) and Perforin-PE (Clone dG9). The T-cell panel consisted of CD3-PECy5 (Clone HIT3a), CD4-BV786 (Clone OKT4), CD8-PECy7 (Clone RPA-T8), CD25-BV510 (Clone M-A251), FOXP3-BV421 (Clone 206D), IFN-γ-BV605 (Clone 4S.B3), IL-4-AF488 (MP4-25D2), IL-9-PE (Clone MH9A4), IL-17A-BV711 (Clone BL168), and IL-22-APC (Clone 2G12A41). The NK cell panel included the following antibodies: CD45-BV605 (Clone HI30), CD3-PECy5 (Clone HIT3a), CD56-FITC (Clone HCD56), CD16-BV785 (Clone 3G8), NKG2D-BV510 (Clone 1D11), NKp30-PE (Clone P30-15), NKp46-BV711 (Clone 9E2), CD107a-BV421 (Clone H4A3), NKG2A-PECy7 (Clone S19004C), CD94-PerCPCy5.5 (Clone DX22), and KIR-APC (Clone Clone HP-MA4).

All antibodies and their respective isotype controls were purchased from BioLegend^®^. All cytometric measurements were performed using LSFortessa II^®^ (BD Biosciences^®^, Franklin Lakes, NJ, USA) and FlowJo^®^ 10.10.0 software.

### 4.5. Assessment of Cytokine Production Using Sandwich ELISA

Both unstimulated and LPS or PMA/Ionomycin stimulated supernatants were thawed and analyzed for cytokine concentrations using the respective ELISA kits, following the manufacturers’ protocols: Human TNF-alpha DuoSet ELISA, Human IL-6 DuoSet, Human IL-2 DuoSet ELISA, Human IL-17 DuoSet ELISA (R&D Systems, Minneapolis, MN, USA), and BD OptEIA™ Human IFN-γ ELISA Set (BD Biosciences).

### 4.6. Assessment of Natural Killer Cell Cytotoxicity

K562 cells, obtained from ATCC (USA), were lentivirally transduced with a pCDH-EF1a-eFly-eGFP plasmid. Enhanced green fluorescent protein (eGFP)-positive cells were sorted using a BD FACSAria™ III Cell Sorter (BD Biosciences^®^). STR profiling was conducted at the LMU Munich Institute for Forensic Medicine to verify the origin of the cell lines, and mycoplasma contamination was ruled out by polymerase chain reaction (PCR). K562 cells were maintained in RPMI medium supplemented with 20% fetal bovine serum (FBS), 2 mM L-glutamine, 100 U/mL penicillin, and 100 µg/mL streptomycin, cultured at 37 °C in a humidified incubator with 5% CO_2_. Cells were cryopreserved at −80 °C or in liquid nitrogen using freezing medium containing 90% FBS and 10% dimethyl sulfoxide (DMSO).

Natural killer cell cytotoxicity (NKCC) was assessed in vitro by co-culturing isolated PBMCs (effector cells, E) with K562 cells (target cells, T) for 4 h at 37 °C and 5% CO_2_ in triplicates. Two effector-to-target (E:T) ratios, 50:1 and 200:1, were evaluated. The cytotoxicity assay was performed using the Bio-Glo™ Luciferase Assay System (Promega^®^, Madison, WI, USA) in accordance with the manufacturer’s instructions. Target cell lysis was calculated as specific lysis using the formula: 1 − (Luminescence of sample/negative control) × 100%.

### 4.7. Statistical Analysis

For statistical analysis, data normality was assessed using the Shapiro–Wilk test. Column statistics, including mean, standard error of the mean (SEM), and quartiles, were calculated using GraphPad Prism. Non-normally distributed data were evaluated using the Kruskal–Wallis test with Dunn’s multiple comparisons post hoc test. A 95% confidence interval was applied, and a *p*-value of <0.05 was considered statistically significant, with significance levels denoted as follows: *p* ≤ 0.05 (*), ≤0.01 (**), ≤0.001 (***), <0.0001 (****). Statistical analyses and graphical visualizations were performed using GraphPad Prism (version 10) and Adobe Illustrator 2020.

## Figures and Tables

**Table 1:** Patient characteristics.

	Reverse-Circadian *n* = 16	Circadian *n* = 38	*p*-Value
Age (years)	15.00 (12.00–19.50)	18.00 (14.75–20.00)	0.0779
Sex			
Female (%)	4 (25)	17 (45)	0.2287
Male (%)	12 (75)	21 (55)	
BMI (kg/m^2^)	24.07 (20.98–27.93)	23.68 (19.59–27.87)	0.6136
BSA * (m^2^)	1.65 (1.51–1.96)	1.70 (1.52–1.98)	0.7984
HDE ** (mg/d)	28.75 (18.75–36.88)	25.00 (17.50–30.00)	0.2013
HDE/BSA ((mg/day)/m^2^)	17.36 (12.30–19.17)	14.46 (11.59–17.36)	**0.0376**

Data are presented as median (IQR). * Body surface area, ** HDE = daily hydrocortisone dose-equivalent dose. Significant differences (*p* < 0.05) are presented in bold letters.

**Table 2:** Glucocorticoid regimen in patients switched from reverse-circadian to circadian treatment.

		Reverse-Circadian Treatment				Circadian Treatment			
	Sex	Age	GC (Type)	GC (mg)	HDE (mg/d)	Age	GC (Type)	GC (mg)	HDE (mg/d)
1	m	11	HC	5–2.5–10	17.5	11	HC	10–2.5–5	17.5
2	m	10	HC	5–2.5–7.5	15	11	HC	7.5–2.5–5	15
3	f	18	HC	10–5–12.5	27.5	18	HC	12.5–5–10	27.5
4	f	14	HC	7.5–7.5–12.5	27.5	15	HC	12.5–7.5–7.5	27.5
5	m	15	HC	10–7.5–12.5	30	15	HC	12.5–7.5–10	30
6	m	14	HC	7.5–7.5–15	30	15	HC	15–7.5–7.5	30
7	f	12	HC	5–5–12.5	22.5	12	HC	12.5–5–7.5	25
8	m	20	HC	7–1–15	23	20	HC	15–1–7	23
9	m	20	HC	10–10–15	35	22	MRHC	15–0–20	35
10	m	20	HC	10–10–15	35	20	MRHC	15–0–20	35
11	m	12	HC	5–5–7.5	17.5	12	MRHC	5–0–10	15

HDE = daily hydrocortisone dose-equivalent dose.

**Table 3:** Comparison of lymphocyte populations in pediatric and adolescent patients with CAH on either reverse-circadian or circadian glucocorticoid replacement regimens.

Lymphocyte Population	Reverse-Circadian *n* = 16	Circadian *n* = 38	*p*-Value
**B-cells**	53.90 (46.30–60.30)	60.85 (48.28–71.80)	0.2010
**Classical monocytes**	0.4300 (0.3000–1.110)	0.5550 (0.2150–1.608)	0.5730
**Intermediate monocytes**	0.3700 (0.2350–0.4400)	0.2850 (0.2100–0.3800)	0.3409
**Non-classical monocytes**	26.50 (19.70–38.95)	23.25 (11.00–31.75)	0.1117
**CD3+ T cells **	75.65 (70.53–79.63)	75.70 (68.05–78.55)	0.4394
**CD4+ T cells**	62.35 (52.03–65.73)	63.80 (55.70–67.60)	0.4684
**IFN-γ+ T_H_-cells (Th1)**	8.160 (5.583–12.68)	9.330 (6.600–13.95)	0.4032
**IL-4+ T_H_-cells (Th2)**	2.510 (1.975–3.405)	2.940 (2.320–3.515)	0.2156
**IL-9+ T_H_-cells (Th9)**	0.2450 (0.1575–0.3300)	0.3200 (0.1900–0.4000)	0.2456
**IL-17+ T_H_-cells (Th17)**	0.4650 (0.2650–0.7950)	0.5500 (0.3750–0.7100)	0.3055
**IL-22+ T_H_-cells (Th22)**	0.4500 (0.3550–0.9775)	0.7300 (0.3900–1.195)	0.3148
**CD4+CD25+ (unstim)**	4.990 (4.125–6.485)	6.780 (5.215–8.945)	**0.0139**
**Foxp3+ Tregs (unstim)**	28.75 (21.90–32.60)	27.10 (21.30–35.75)	0.8470
**CD8+ T cells**	29.40 (26.23–35.90)	28.50 (24.35–33.25)	0.3614
**IFN-γ+ T_C_-cells (Tc1)**	24.10 (18.60–31.60)	28.20 (19.60–39.10)	0.3739
**IL-4+ T_C_-cells (Tc2)**	1.535 (1.020–3.053)	2.030 (0.9300–3.180)	0.7481
**IL-9+ T_C_-cells (Tc9)**	2.200 (1.003–4.040)	1.960 (1.050–3.625)	0.9503
**IL-17+ T_C_-cells (Tc17)**	0.4550 (0.2900–1.535)	0.7300 (0.2900–1.650)	0.6836
**IL-22+ T_C_-cells (Tc22)**	0.9350 (0.7700–1.895)	1.230 (0.8150–1.805)	0.8292
**NK cells (CD3-CD56+) **	17.15 (11.40–21.68)	18.50 (11.25–26.68)	0.6026
**CD56brightCD16dim (unstim)**	1.730 (1.388–2.885)	2.790 (1.463–4.155)	0.1728
**CD56dimCD16bright (unstim)**	20.65 (8.915–28.68)	18.65 (7.395–28.40)	0.7393
**CD94+ NK cells**	47.35 (42.23–57.20)	51.70 (40.63–58.85)	0.7575
**CD107a+ NK cells**	45.05 (35.30–49.83)	58.70 (46.55–65.98)	**<0.0001**
**KIR+ NK cells**	19.20 (11.78–21.08)	18.05 (12.48–21.18)	0.7821
**NKG2A+ NK cells**	36.45 (31.25–42.43)	37.30 (26.00–45.40)	0.7778
**NKG2D+ NK cells**	17.55 (10.39–28.13)	22.85 (15.20–30.55)	0.2016
**NKp30+ NK cells**	46.45 (31.80–61.88)	33.10 (25.50–43.73)	**0.0189**
**NKp46+ NK cells**	13.70 (9.675–21.03)	15.30 (11.13–24.78)	0.4726

Presented are the median (IQR) values of different leucocyte subsets as a percentage of the parent population of pediatric and adolescent patients with CAH on either reverse-circadian or circadian glucocorticoid replacement regimens. *p* values refer to the unpaired *t*-test (in case data were normally distributed) or Mann–Whitney test (for non-normally distributed data) with *p* < 0.05 considered as clinically significant (marked in bold).

**Table 4:** Comparison of lymphocyte populations in pediatric and adolescent patients with CAH on reverse-circadian treatment as well as after switch to circadian GC replacement.

Lymphocyte Population	Reverse-Circadian *n* = 11	Circadian *n* = 11	*p*-Value
**B-cells**	53.90 (49.70–58.40)	63.30 (56.70–70.10)	0.1230
**Classical monocytes**	0.5500 (0.3800–1.250)	0.4600 (0.2000–1.660)	0.8467
**Intermediate monocytes**	0.3800 (0.2300–0.4400)	0.3700 (0.2850–0.5050)	0.9102
**Non-classical monocytes**	26.00 (19.70–37.35)	18.70 (13.55–26.30)	**0.0255**
**CD3+ T cells **	78.40 (70.60–80.70)	75.70 (68.90–80.90)	0.1868
**CD4+ T cells**	64.40 (57.80–67.00)	64.70 (59.80–67.00)	0.8311
**IFN-γ+ T_H_-cells (Th1)**	8.030 (5.550–12.30)	8.520 (4.400–14.40)	0.5771
**IL-4+ T_H_-cells (Th2)**	2.530 (2.150–3.430)	2.610 (2.230–3.440)	0.8984
**IL-9+ T_H_-cells (Th9)**	0.2600 (0.2000–0.3000)	0.2400 (0.1900–0.3300)	0.9824
**IL-17+ T_H_-cells (Th17)**	0.4500 (0.2600–0.5600)	0.4600 (0.3600–0.7400)	**0.0195**
**IL-22+ T_H_-cells (Th22)**	0.4400 (0.3700–0.7600)	0.4200 (0.3200–1.250)	0.5918
**CD4+CD25+ (unstim)**	4.930 (4.050–6.540)	5.000 (3.620–6.930)	0.2715
**Foxp3+ Tregs (unstim)**	29.70 (21.90–32.70)	24.90 (21.70–35.70)	0.5864
**CD8+ T cells**	29.10 (23.20–35.60)	28.50 (26.60–35.70)	0.9160
**IFN-γ+ T_C_-cells (Tc1)**	22.40 (18.00–28.20)	18.90 (16.30–38.10)	0.7279
**IL-4+ T_C_-cells (Tc2)**	1.470 (0.9900–3.320)	1.310 (0.7200–3.500)	0.8984
**IL-9+ T_C_-cells (Tc9)**	1.690 (0.9300–4.330)	1.900 (1.050–4.400)	0.8984
**IL-17+ T_C_-cells (Tc17)**	0.4100 (0.2900–1.560)	0.2800 (0.1800–1.890)	0.5352
**IL-22+ T_C_-cells (Tc22)**	0.8900 (0.7700–1.670)	0.8400 (0.5900–2.120)	0.9658
**NK cells (CD56+ CD3-)**	23.30 (13.30–29.50)	22.10 (13.40–29.80)	0.8539
**CD56brightCD16dim**	1.720 (1.470–2.920)	1.880 (0.8300–3.430)	0.9543
**CD56dimCD16bright**	20.80 (11.00–27.70)	20.50 (12.10–31.60)	0.9783
**CD94+ NK cells**	51.30 (43.20–57.90)	54.50 (37.10–66.20)	0.4469
**CD107a+ NK cells**	42.20 (38.50–49.90)	44.50 (43.00–56.30)	0.1640
**KIR+ NK cells**	20.90 (12.00–27.20)	18.70 (13.80–21.40)	0.7646
**NKG2A+ NK cells**	38.80 (30.60–42.70)	34.30 (21.80–45.40)	0.7638
**NKG2D+ NK cells**	16.60 (9.690–28.40)	23.40 (17.90–36.60)	**0.0420**
**NKp30+ NK cells**	44.10 (30.70–64.20)	40.00 (29.00–60.30)	0.0609
**NKp46+ NK cells**	16.40 (11.70–21.50)	15.10 (11.50–21.20)	>0.9999

Presented are the median (IQR) values of different leucocyte subsets as a percentage of the parent population of pediatric and adolescent patients with CAH on reverse-circadian treatment as well as after the switch to circadian GC replacement. *p* values refer to the paired *t*-test (in case data was normally distributed) or Wilcoxon signed rank test (for non-normally distributed data), with *p* < 0.05 considered as clinically significant (marked in bold).

## Data Availability

The datasets generated during and/or analyzed during this study are not publicly available but are available from the corresponding author upon reasonable request.
